# Student Continuity with Patients: A System Delivery Innovation to Benefit Patient Care and Learning (Continuity Patient Benefit)

**DOI:** 10.3390/healthcare3030607

**Published:** 2015-07-22

**Authors:** Ann N. Poncelet, J. Nicky Hudson

**Affiliations:** 1 Department of Neurology, University of California, San Francisco, 400 Parnassus Avenue, Box 0348, UCSF, San Francisco, CA 94143, USA; 2 Department of Rural Health, University of Newcastle, Tamworth Education Centre, 114–148 Johnston St., Tamworth, NSW 2340, Australia; E-Mail: nicky.hudson@newcastle.edu.au

**Keywords:** longitudinal integrated clerkship, patient experiences, patient-centered care, continuity, medical education

## Abstract

Medical education is continuing to evolve to meet the healthcare needs of the future. The longitudinal integrated clerkship (LIC) model is an important innovation in medical education. It has in its vision and structure “patient- and learner-centered education”, using longitudinal relationships between patients and students as a foundational element in its design. LIC students have shown more patient-centered attitudes and behaviors that persist after medical school. They remain connected with the patient experience of care, which supports empathy and student moral development. The time that LIC students spend acting independently with patients also supports the development of higher order clinical and cognitive skills and professional identity formation. Student participation in a more meaningful way in the care of their patients promotes patient wellbeing, and helps patients with transitions of care, communication and preventative care. Patients report feeling empowered to be more active agents in their own care and feel an accountability and pleasure in the training of new physicians. Focusing on the patient/student relationship as a foundational element of clinical education has meaningful benefits to the patient and student with the potential to improve patient care directly and in the future, as these students become physicians.

## 1. Introduction

Medical education is continually evolving to meet the healthcare needs of the future. One important and relatively recent innovation is the longitudinal integrated clerkship (LIC), a model of clinical education with continuity as its organizing principle [1]. The essence of this clerkship learning model is continuity relationships between patients and medical students. In an LIC, students spend the majority of the clinical year (usually 6–12 months) working with a preceptor or preceptors and a panel of patients, and through these longitudinal relationships meet the core learning goals of the clinical year [1]. This model of clinical training has been implemented in the UK, Australia, Canada, and the United States [1,2,3,4,5]. The first LIC was launched over 40 years ago but was rare until the last 10 years. Currently, there are 45 known LIC programs worldwide (Consortium of Longitudinal Integrated Clerkships Research Collaborative).

LICs tend be in one of two general structures, depending upon the context in which they are housed. They are either community-based LICs (the student is based in general practice and is mentored by a general practice (GP)/family physician) or in a more specialized setting such as an urban academic medical center (the student is based in parallel ambulatory care practices mentored by primary care and specialist physicians) [6]. In the former, the student spends the entire LIC in the generalist practice seeing patients with the primary care physician and other generalists as present, such as a surgeon or obstetrician in the clinic or local hospital [5,7]. In the parallel model, the student may spend every Monday morning with a pediatrician, Monday afternoon with a surgeon, Tuesday morning with an internist and so on over the course of the year [3,4]. The student acquires through these sessions a panel of patients who they follow where ever they go (e.g., specialty consultations, emergency room, operating room, *etc.*). In both models, continuity with patients is a key element of the education.

Continuity and long-term relationships in medical education are important for learning and professional identity formation [8]. Educational continuity as a structure for core clinical training allows for continuity of care, continuity of curriculum and continuity of supervision. Longitudinal relationships between students, supervisors and patients are crucial for what has been termed “patient-centered medical education” where the patient and the patient’s experience of illness and medical care become a key driver of learning. Bleakley and Bligh suggest that “patient-centered medical education” changes the relationship between the student and patient from the patient being an object of learning to a partnership with collaborative knowledge building for both the student and patient [9]. Shared experience over time enables the patient to teach patient-centeredness directly to the student. The medical educator in this context has a guiding role with the primary learning generated between the student and the patient.

Continuity relationships between patients and their health providers are known to result in many benefits for patients, for example, better coordination of care and communication of values [10,11], mitigation of the information asymmetry between patients and providers [12] and patients experience more empathy and holistic care [11]. Continuity alone is not enough for these benefits, but allows for the formation of a strong patient-physician relationship [10,11]. When a student experiences continuity of supervision by clinicians with long-term and strong relationships with their patients, the student can participate and develop a sense of belonging in the patient-care team(s). Student participation is legitimized by the trust these patients have in the supervising doctors, but over time students develop their own long-term relationships with patients and become a more central, rather than a peripheral member of the care-team [13]. There is increasing evidence that continuity with the opportunity for patients and students to develop meaningful relationships is mutually beneficial to both. Benefits to patients occur on two levels: (1) direct benefit to the patient from working with the student [13,14,15], and (2) creation of skilled physicians who are better trained to provide patient-centered care [16]. An additional benefit in some settings is recruiting future physicians to rural and underserved areas [17,18]. Benefits to the students include having more responsibility and an authentic role in patient care, patient-centered communication skills, more preparedness in higher-order clinical and cognitive skills, support of professional identity formation and being more effective within a system [19,20,21]. This evidence comes mostly from preceptors and students with limited evidence from patients. The purpose of this review is to explore the longitudinal integrated clerkship as a delivery system innovation to improve patient care and the patient experience of care.

## 2. Methods

There is a growing published literature on the outcomes of long-term student-patient relationships. These studies include patient, supervisor and student interviews with qualitative analysis, observational work-study, observation in the clinical setting, validated surveys, and excerpts from student clinical logs. The literature was searched for evidence of how the LIC innovation impacts on patients and their care.

### 2.1. Search Strategies

JNH and ANP carried about a search of PubMed and Medline, CINAHL and EMBASE databases between 1 April and 19 April, 2015 using the search terms “longitudinal AND clerkships AND patients”. The initial search was run over Medline and PubMed concurrently, and then on the remaining databases. The latter two databases revealed no new publications than those identified on PubMed and Medline. JNH also searched the contents of three of the major medical education journals (Academic Medicine, Medical Education and Medical Teacher) using the same search terms. No new publications were identified. Several references, not found on the above search, were known to the authors (as co-authors of these book chapters) [6,22].

### 2.2. Inclusion and Exclusion Criteria

A longitudinal integrated clerkship is defined as characterized by being the central element of clinical education whereby medical students (1) participate in the comprehensive care of patients over time; (2) participate in continuing learning relationships with these patients’ clinicians; and (3) meet the majority of the year’s core clinical competencies, across multiple disciplines simultaneously through these experiences [1]. Papers included in this review had to meet this criteria but were not limited by setting (rural *versus* urban, placement in generalist practice *versus* multiple discipline specific preceptors). Table 1 summarizes the inclusion and exclusion criteria.

**Table 1:** Inclusion and Exclusion Criteria *.

Inclusion	Exclusion
Medical students	Health professional students other than medical
Placements longer than 24 weeks	Placements of less than 24 weeks
Student access to and continuity of patient population in same location and/or continuity of preceptor/supervisor and meets definition for LIC	No continuity of patients and location and/or preceptor/supervisor and/or does not meet definition for LIC
Evaluation data relating to effectiveness of placements	No evaluation data relating to effectiveness of placements
In English	Not in English

* Modified from Thistlethwaite *et al.* 2013 [23].

### 2.3. Strengths and Limitations of Evidence

The data on the impact of the LIC initiative on patients and their care is limited due to the paucity of literature on this theme to date. However, the current evidence has emerged from qualitative studies of appropriate rigor, from two different models of LIC, and from different international settings. This supports the claim that patients perceive they gain from continuity of the student-patient relationship.

## 3. Results and Discussion

### 3.1. Benefit to Patients

#### 3.1.1. Direct Benefit to Patient Care

LIC students feel they have a role in improving patient care, particularly bridging gaps in the health care system, facilitating transitions of care, improving communication between providers and the patient, and providing comfort and support to the patient.

“*Without me I can confidently say this illiterate, non-English-speaking patient, even with his very supportive and involved family, would have fallen through the cracks. The number of appointments and communications and miscommunications would have been so numerous…that he probably would have just stopped showing up.*”

—CIC* LIC Student MG [14]

“*We both [patient and student] benefited from my long-term relationship with the patient. The patient was a 45–65 year old male with gastrointestinal upset on a background of chronic alcohol abuse, cirrhosis and chronic pancreatitis. This man had been lost to the system and was on a slippery downward slope. I saw him on a regular basis. On the first consultation he was very reserved and quite defensive when questioned about his alcohol consumption, over the year we managed to significantly reduce his alcohol consumption, gain better control of his diabetes and managed to get his to have a colonoscopy, which was a massive task for him.*”

—UoW** LIC student [22]

Students as “value added” is mirrored in the patient perceptions of working with students longitudinally and increases with the number of contacts with the students over time and across settings [15]. An interview study of patients working with LIC students in an urban academic medical center revealed that patients experienced improved wellbeing and feeling supported as a result of working with LIC students over time [15]. They experienced the students to be more patient-centered than other members of the health care team and appreciated the humanistic quality of communication with their students. They described the importance of the student getting to know them as a person and treating the person, not just the disease.

“*I think literally it was just him asking me questions, seeing how I was doing and genuinely listening. Asking about my wellbeing, as opposed to just checking my pulse and everything else that was happening to me.*”

—UCSF*** LIC patient 531 [15]

*: Cambridge Integrated Clerkship, Harvard Medical School.
**: Graduate School of Medicine, University of Wollongong (UoW).
***: University of California, San Francisco (UCSF).

“*When you go to see a physician you’re lucky to get the first 10–20 s of the doctor’s attention. I always go in with a list of questions, and I still have a degree of anxiety because I know I won’t get through all the questions before the doctor’s out of there. So it was lovely to have a student take his time and do a good job, and listening, and I think it’s a terrific thing to do.*”

—UCSF LIC patient 674 [15]

Patients who attended general practices that hosted LIC students in rural Australia expressed similar sentiments when interviewed about their year-long experiences with LIC students.

“*Long-term is a good idea rather than short-term. You get that personal touch because they [students] have got time to get to know exactly what’s going on.*”

—UoW rural LIC patient [13]

“*One of the students asked me how it affects my life or how I’ve coped and I think it is very important for a patient.*”

—UoW rural LIC patient [13]

“*She [the student] has basically been managing me since I first got out of hospital…she was another doctor but she’s been with me through my whole case with my treating GP…and every time he (GP) would come and consult and it saves a lot of time it’s a better quality of service, cos I was getting two doctors for the price of one.*”

—UoW rural LIC patient [22]

In the LIC model, patients also describe students functioning as a bridge with patient’s physicians and providing a physician-like contribution to their care. Students provide patient education, answering and researching questions, explaining medical issues and/or procedures, discussing test results and making medical recommendations. They help to coordinate care such as scheduling appointments and helping patients to navigate the medical system. They serve an important role communicating with the patient, between the patient and the physician and between physicians.

“*Dealing with the ordeal of having cancer and going through treatment at a hospital like UCSF, which is very busy, can be kind of overwhelming at times. I kind of looked to him as a friendly face, sort of a liaison between the doctor and the patient.*”

—UCSF LIC patient 504 [15]

“*She would make phone calls for me, tell me where to show up and tell me what time and who would meet me there. Sometimes she would even meet us over there at the hospital. She facilitated a lot of us getting into the hospital and making things more comfortable.*”

—UCSF LIC patient 596 [15]

“*We had a great talk about everything and I asked him questions and he answered them very professionally. The [student] fulfilled my expectations.*”

—UoW rural LIC patient [13]

“*He [Dr.] went right through with her [student], the questions that she asked, what she had decided should have been the treatment and gave her some suggestions but always while I was present. I appreciated that…yes felt part of the decision-making process. Nothing was hidden, that’s really important*.”

—UoW rural LIC patient [13]

Hudson and coworkers reported that in a LIC program in regional, rural and remote settings in Australia [13], patients felt “empowered” by their longitudinal relationship with LIC students. Patients, as well as students, became part of the community of practice in their local general practice “teaching microsystem”. Patients viewed the clerkship learning environment as patient- and student-centred, emphasizing that the extended patient-student-doctor relationship triad was important in facilitating active participation by patients as well as students. Patients gained greater respect for their doctors as they observed them mentoring and supervising the students, and reported that they, as well as students, underwent identity transformation over time. There was reciprocity between all participants in the learning triad with each assuming the role of learner or teacher according to the learning situation. Patients felt like collaborators in the team attending to their health and well-being. As the students grew in confidence and competence over the clerkship year, patients reported that LIC students value-added to the care they received. They also improved patient access to care in these settings with health workforce shortage. Thus there were benefits to the system, as well as to individual patients.

“*I find medical students are just…they’re willing to learn…they’re just a lot more aware of the patient I think sometime. I met her at the beginning of my pregnancy. I went through a few things…you’d come to visit again and she’d be there. It was kind of like she was growing with me…if that makes sense.*”

—UoW rural LIC patient [22]

“*My own GP said it was something he doesn’t see very often. He wanted to give the student an experience of seeing how she went diagnosing. He had a quiet word with me. It was very valuable for all of us because it was me talking to the young student doctor, but having the other doctor behind. He and I knew what the diagnosis was.*”

—UoW rural LIC patient [13]

“*The patient is always involved. It’s not like the doctor takes the student out of the room…the student is there and actually gets involved. I think it’s good because they’re learning a lot, plus the patient is learning trust…some of these students might be here at the surgery later on in life.*”

—UoW remote LIC patient [13]

“*What I’ve observed with both doctors…is the way the doctors support the students…for me it enhances my relationship with the doctor*.”

—UoW rural LIC patient [13]

#### 3.1.2. Creation of Patient-Centered Physicians

Medical education attracts students who are altruistic and care deeply about patients. Despite these strong qualities, there is evidence that students suffer ethical erosion over the four years of medical school and fail to further develop their moral reasoning [24,25,26]. They also suffer a loss of patient-centeredness shifting to a more doctor-centered view [27]. A major factor in this loss is thought to be the “hidden curriculum” which is latent or implicit social processes that influence a student’s behaviour [28]. For example, students know when they are in the first year that they should introduce themselves to each patient. However, on a busy service they may observe the more senior trainees and/or faculty failing to do so when on clinical rounds. Without being aware of it, a student may internalize this behaviour and emulate it as the norm in a busy clinical setting.

Continuity between students, patients and supervisors is proposed as a way to mitigate the hidden curriculum and promote patient-centered behaviors and attitudes in medical students [8,29]. Patient and student perspectives suggest the LIC model does support patient-centered attitudes and behaviors [13,14,15]. LIC students also report feeling more confident when confronted with ethical dilemmas [21]. Using the physician-patient orientation scale (PPOS) which is a validated measure of patient-centered attitudes, Krupat and colleagues showed that traditional medical students from Harvard Medical School suffered the previously described erosion of patient centeredness in contrast to the LIC students where it was preserved [30]. More recently, Gaufberg and co-authors showed LIC graduates from the Harvard Medical School Cambridge Integrated Clerkship not only emerged from their clerkship year with a higher degree of patient-centeredness but that this endures over time [16].

LIC students describe a rich array of benefits from longitudinal relationships with patients to their development as physicians [14,19]. These benefits include creating a strong interpersonal bond between student and patient and getting to know the patient in the context of family and community. The LIC students feel these relationships promote empathy, foster a sense of duty, commitment and ownership. They report seeing the patient as a person, creating a student-patient team, and caring for rather than distaining “difficult” patients. These are the professional attributes that medical educators aim to develop so learners become patient-centered physicians.

“*They [LIC students] are just a lot more aware of the patient I think sometimes.*”

—UoW rural LIC patient [13]

“*I believe very strongly that the profound sense of my unique longitudinal experience with him bonded us together and required me to take responsibility in a way I would not traditionally be asked to do.*”

—CIC LIC Student AH [14]

“*They are polite and they’re thinking of the patient…they are thinking of you and make you comfortable. The students listened to him [6-year-old grandson]. He was only six but he knew more than me and his mother knew because he’d been around with the doctors…he was telling them [students]…they were asking him questions...they weren’t talking to him like he was way down here and they were way up here. It was helpful for my grandson too.*”

—UoW remote LIC Patient Grandmother [22]

#### 3.1.3. Addressing Workforce Shortages

A number of LIC programs were created in part to address a workforce shortage, particularly in rural areas. The argument is that students trained in urban academic medical centers are unlikely to choose rural practice after graduation. Longitudinal placement of students in rural or other community-based settings is thought to be one way of supporting students to choose careers in these settings. Rural and community-based LICs such as the University of Minnesota Rural Physician Associate Program (RPAP) and the Parallel Rural Community Clerkship (PRCC) at Flinders University School of Medicine have shown success in recruiting graduating medical students to practice in rural communities [17,18,21]. Rural patients in Australia acknowledged that the LIC initiative was a good idea as they were always short of doctors in their rural community. Clearly having students contributing to patient-care and then returning to rural practice as physicians is a great outcome for access to health care in these communities.

“*It’s maybe taken a bit of a load off her [doctor]…the doctors do work pretty hard.*”

—UoW rural LIC patient [13]

“*I think it definitely a positive and hopefully when they graduate, it gets them back into the country.*”

—UoW rural LIC patient [13]

“*It does contribute to the community…we’ve got additional doctors in a way.*”

—UoW rural LIC patient [13]

### 3.2. Benefit to Students

#### 3.2.1. Student Role and Responsibility

The LIC model results in students spending more time with patients compared to block clerkship students. An observation study of LIC students compared to inpatient block clerkship students from 3 medical schools showed the LIC students at the end of the year spend almost 50% of their time with patients, half of which is performing independently compared to the block students who only spent 20% of their time at the bedside, 7% of which is performing independently [20]. LIC students and patients both report the LIC student taking on more responsibility for patient care and having an authentic role in their care [14,15,19,21,31]. They feel ownership of their patient’s care and refer to their patients as “my patient” as opposed to “a” or “the” patient [31]. They feel valued by the patients as opposed to being a burden.

In contrast to traditional students, the LIC students describe functioning in a doctor-like role by the end of the clerkship [19,31]. The patients and supervisors in the LIC programs provide an invitational quality for the student to actively participate in the patient’s care. Students report better access to patients, seeing a broader range of presenting conditions, and learning through experience about the course of illness [21]. Continuity with patients also allows students to share information about patients across health care settings [19].

“*I was able to not only see him in the emergency department, but see him a couple of weeks later in the rheumatology clinic. Immediately, he saw me and he recognized me. He knew that was helping to take care of him in the ED. I was immediately welcomed into his visit and I was even able to share some of his history with the rheumatologist who was seeing him…I felt really useful.*”

—LIC student (three school study) [19]

“*You go in and see a patient that you’ve seen many, many times who knows you very well and you know them very well. You’re checking up on their longstanding diabetes and seeing if they’re still taking their medications, or if they’ve tried walking the extra block or whatever we were working on. That really gave me a sense of being a physician.*”

—LIC student (three school study) [19]

#### 3.2.2. Clinical Skills and Professional Development

Students in LICs develop strong patient centered communication skills, demonstrate understanding of the psychosocial contributions to medicine, and report more preparedness in higher-order clinical and cognitive skills in comparison to students in traditional block rotations [21]. There is a significant impact in the students’ professional development including understanding their own limits, having a greater confidence in dealing with uncertainty, utilizing reflective practice, and being self-directed. Improved clinical skills and support for professional development ultimately have the potential to positively impact future patient care as these students enter the workforce. Patients themselves have highlighted the benefits of learner participation in practice (instead of book learning) to prepare the medical students for patient care.

“*You can read anything out of a book…totally different…they [student] are getting to see people like me but they’re interacting with all age groups.*”

—UoW remote LIC patient [22]

## 4. Conclusions

When continuity between students and patients is built into the structure of clinical training, the result is a powerful positive impact on student learning and benefits patient perceptions of care with the promise of benefits to patient care. Evidence continues to grow on the positive impact of this symbiotic model of medical education, which enables the student to create a positive synergy between the patient and the physician through longitudinal relationships. This model has the potential to improve direct patient care as well as the quality of physicians trained in this model.
